# Pancreatic family history does not predict disease progression but connotes alcohol consumption in adolescents and young adults with acute pancreatitis: Analysis of an international cohort of 2,335 patients

**DOI:** 10.3389/fmed.2022.801592

**Published:** 2022-09-12

**Authors:** Márk Félix Juhász, Nelli Farkas, Andrea Szentesi, Andrzej Wedrychowicz, Andreia Florina Nita, Natália Lásztity, Alexandra Tészás, István Tokodi, Áron Vincze, Bálint Eross, Ferenc Izbéki, László Czakó, Mária Papp, Péter Hegyi, Andrea Párniczky

**Affiliations:** ^1^Institute for Translational Medicine, Szentágothai Research Centre, Medical School, University of Pécs, Pécs, Hungary; ^2^Centre for Translational Medicine, Semmelweis University, Budapest, Hungary; ^3^Department of Medicine, Centre for Translational Medicine, University of Szeged, Szeged, Hungary; ^4^Department of Pediatrics, Gastroenterology and Nutrition, Faculty of Medicine, Jagiellonian University Medical College, Krakow, Poland; ^5^Department of Paediatrics, Grigore Alexandrescu Emergency Hospital for Children, Bucharest, Romania; ^6^Department of Pediatrics, Szent János’s Hospital and North Buda Unified Hospitals, Budapest, Hungary; ^7^Department of Paediatrics, University of Pécs Clinical Centre, Pécs, Hungary; ^8^Szent György University Teaching Hospital of Fejér County, Székesfehérvár, Hungary; ^9^Division of Gastroenterology, First Department of Medicine, Medical School, University of Pécs, Pécs, Hungary; ^10^Division of Pancreatic Diseases, Heart and Vascular Center, Semmelweis University, Budapest, Hungary; ^11^Department of Medicine, University of Szeged, Szeged, Hungary; ^12^Division of Gastroenterology, Department of Internal Medicine, University of Debrecen, Debrecen, Hungary; ^13^Heim Pál National Pediatric Institute, Budapest, Hungary

**Keywords:** acute pancreatitis, family history, harmful alcohol consumption, genetic, recurrent pancreatitis

## Abstract

**Background:**

In pediatric acute pancreatitis (AP), a family history of pancreatic diseases is prognostic for earlier onset of recurrent AP (ARP) and chronic pancreatitis (CP). No evidence supports the same association in adult-onset pancreatitis. Age-specific reasons for familial aggregation are also unclear. We aimed to examine the prognostic role of pancreatic family history for ARP/CP and observe possible underlying mechanisms.

**Methods:**

We conducted a secondary analysis of the Hungarian Pancreatic Study Group’s (HPSG) multicenter, international, prospective registry of patients with AP, both children and adults. We compared the positive family history and the negative family history of pancreatic diseases, in different age groups, and analyzed trends of accompanying factors. Chi-square and Fisher exact tests were used.

**Results:**

We found a higher rate of ARP/CP in the positive pancreatic family history group (33.7 vs. 25.9%, *p* = 0.018), peaking at 6–17 years. Idiopathic AP peaked in childhood in the positive family history group (75% 0–5 years) and was consistently 20–35% in the negative group. A higher rate of alcohol consumption/smoking was found in the positive groups at 12–17 years (62.5 vs. 15.8%, *p* = 0.013) and 18–29 years (90.9 vs. 58.1%, *p* = 0.049). The prevalence of diabetes and hyperlipidemia steadily rose with age in both groups.

**Conclusion:**

Positive family history most likely signifies genetic background in early childhood. During adolescence and early adulthood, alcohol consumption and smoking emerge—clinicians should be aware and turn to intervention in such cases. Contrary to current viewpoints, positive pancreatic family history is not a prognostic factor for ARP and CP in adults, so it should not be regarded that way.

## Introduction

Acute pancreatitis (AP) is the sudden onset inflammation of the pancreas, elicited by gallstones or alcohol consumption in 70–80% of adult cases (1). In pediatric AP, the picture is much more diverse: biliary obstruction and drugs account for half of the cases, other etiologies are below 5–10% and the rate of idiopathic cases is higher, around 20–30% as opposed to the 10% found in adults (2–5). In idiopathic cases, there is a higher possibility of inherited genetic alterations in the background, posing a constant and unamendable risk factor, thus increasing the likelihood of and speeding up progression toward acute recurrent pancreatitis (ARP), chronic pancreatitis (CP), and pancreatic cancer (PC) (6). Guidelines recommend that after a second idiopathic AP episode, children should go through genetic testing (7), and adults should receive genetic counseling (not necessarily testing) (8). Therefore, genetic background is often established late and often missed altogether—especially in adults or when other etiologies are present. There is however an easily assessable factor that could point towards genetic predisposition, and be useful in such cases: positive pancreatic family history.

The importance of gathering pancreatic (AP, ARP, CP, PC, etc.) family history is well-established in pediatric pancreatitis, with a family history of AP and CP being strongly associated with earlier ARP and CP onset (9), and the guidelines recommend genetic testing after a single idiopathic episode in case family history is present (7). Adult CP guidelines also strongly recommend assessment (100% agreement) (10); however, there is scarce evidence supporting this recommendation—we failed to identify any clinical studies examining the connection between ARP, CP, and pancreatic family history. Recent years’ literature on ARP and CP highlights the importance of both the identification of risk factors for disease progression and uncovering underlying mechanisms (11, 12). Thus, even though assessing family history is uncomplicated, examining it poses two major points of importance: observing whether it is a risk factor for disease progression in adults; and mapping associations with possible explanatory factors, to reach a greater understanding of AP, ARP, and CP.

Our aim was to examine associations between pancreatic family history, ARP and CP rates, idiopathic etiology, and risk factors of AP in different pediatric and adult age groups. Our findings suggest that (1) family history should not be used as a prognostic factor for ARP or CP in adults, (2) familial aggregation is mostly due to genetic factors in early childhood, and (3) due to increased alcohol consumption and smoking in adolescence and early adulthood.

## Materials and methods

### Study design and data collection

This study is a secondary analysis of the international, multicenter, prospective AP registry maintained by the Hungarian Pancreatic Study Group (HPSG). Between 2012 and 2019, 2,559 episodes of AP were enrolled in the registry. The diagnosis was established according to the International Association of Pancreatology/American Pancreatic Association (IAP/APA) guidelines (8). A list of study sites can be found in our Supplementary material (Supplementary Figure 1 and Supplementary Table 1). A rigorous, four-tier quality control system was applied to ensure the accuracy of these data. For more details on this system, see the previous publication from this registry by Párniczky et al. (13).

### Participants

In the present analysis, both adult and pediatric AP patients with available data on the presence/absence of pancreatic family history—such as AP, CP, ARP, autoimmune pancreatitis (AIP), and PC—were included (2,335 patients, with 2,470 prospectively collected episodes of AP). In our analyses, we compared patients with a negative pancreatic family history to patients with a positive pancreatic family history for AP, CP, ARP, AIP, or PC. To observe age-specific changes in our observed variables, we divided the cohort into age-based subgroups: 0–5, 6–11, 12–17, 18–29, 30–41, 42–53, 54–65, and 66 years. To avoid arbitrary threshold selection, we adhered to the following rhetoric: we planned to divide children into as many equal age-interval groups as possible; since two groups are not yet informative and four resulted in very low participant numbers, we decided to use three equal age intervals. In the case of adult participants, we doubled this interval (from 6 to 12 years) since changes are not as swift as in childhood. We intended to maintain the 6-year interval in early adulthood; however, the 18–23 group would have had zero patients with positive pancreatic family history.

### Variables

All analyzed variables—such as demographical data, data on comorbidities, smoking, alcohol consumption, complications, severity, etiology, and number of episodes—are provided in the data quality table in our Supplementary material (Supplementary Table 2). We adhered to the revised Atlanta criteria in determining the complications and severity of AP: cases were considered mild if no local complications or organ failure occurred, moderate if local complications and/or organ failure lasting less than 48 h occurred, and severe if organ failure persisted beyond 48 h (14). While the prospective data collection period only covers 8 years, a detailed personal medical history was taken, especially regarding the pancreatic disease, and we accounted for these data in determining the presence of ARP and the number of episodes. Patients were assessed to have “hyperlipidemia” if their AP was caused by hypertriglyceridemia or if they were diagnosed with a non-transient dyslipidemia.

We compared our examined cohort to the entirety of the AP cases enrolled in our registry to see whether our analyzed population is representative of the average AP experiencing population. Since almost all patients (96.6%) had data on the presence of pancreatic diseases in the family, our cohort was representative in terms of age, gender, AP severity, mortality, length of hospitalization, and etiology (Supplementary Figure 2).

### Statistical analysis

In the case of categorical variables, we calculated event number and percentage of the total and mean and standard deviation (SD) for continuous data. To test for statistically significant differences between groups, the chi-squared or Fisher’s exact tests were applied for categorical, Student’s t-test for normally distributed continuous, and the Mann-Whitney U test for non-normally distributed continuous variables, with an alpha value of 5%. Statistically significant *p*-values (*p*) appear in bold.

### Ethical approval

The Scientific and Research Ethics Committee of the Medical Research Council granted the ethical approval for this registry in 2012 (22254–1/2012/EKU). The institution’s human research committee approved the protocol for the registry before initiating participant enrolment. We are in compliance with the Declaration of Helsinki, reaffirmed in 2013. All patients provided their written, informed consent in case of participation.

### Study reporting

This study was reported according to the “Strengthening the Reporting of Observational Studies in Epidemiology” (STROBE) statement (15).

## Results

### Participants

Table 1 shows the characteristics of enrolled participants. A total of 2,335 patients were analyzed, of which 196 (8.4%) had a positive pancreatic family history. These patients were younger at the time of their first enrolment in our registry, and idiopathic AP etiology was more common. Mild disease course occurred significantly more often in the case of the first registered AP episode and any prospectively collected episode that belonged to a positive pancreatic family history group as well. The total number of episodes/persons (accounting not only for registry enrolments but also for episodes in medical history) was significantly higher in the positive pancreatic family history group.

**TABLE 1:** Characteristics of participants.

	Positive pancreatic family history	Negative pancreatic family history	*p*
Number of patients	196	2,139	
Female sex; *n* (%)	87 (44.4)	951 (44.5)	*0.984*
Age at first enrolment; years *mean* ±*SD*	49.2 ± 20.4	55.6 ± 18.2	***<0.001***
AP etiology, first enrolment; *n* (%)			
Biliary	66 (33.7)	868 (40.6)	*0.059*
Alcoholic	31 (15.8)	393 (18.4)	*0.374*
Hypertriglyceridemia	8 (4.1)	70 (3.3)	*0.546*
Any combination of these three	15 (7.7)	92 (4.3)	***0.032***
Idiopathic	51 (26.0)	418 (19.5)	***0.030***
Other	25 (12.8)	298 (13.9)	*0.648*
AP severity, first enrolment; *n* (%)			
Mild	154 (78.6)	1522 (71.2)	***0.027***
Moderate	33 (16.8)	510 (23.8)	***0.026***
Severe	9 (4.6)	107 (5.0)	*0.800*
AP severity, any registered episode; *n* (%)			
Mild	168/216 (77.8)	1610/2254 (71.4)	***0.047***
Moderate	39/216 (18.1)	533/2254 (23.6)	*0.063*
Severe	9/216 (4.2)	111/2254 (4.9)	*0.621*
AP episodes / person; *mean* ±*SD*	1.74 ± 1.86	1.48 ± 1.29	***0.010***

AP, acute pancreatitis; n, number; SD, standard deviation; %, percentage; *p*, *p*-value.

Regarding the age distribution of positive family history, among adults, the observed rate was steadily around 8% (6.4–9.4%), but it was considerably higher in the case of children, peaking at 6–11 years (40.0%; Supplementary Figure 3).

### Pancreatic family history, acute recurrent pancreatitis, and chronic pancreatitis

**FIGURE 1:**
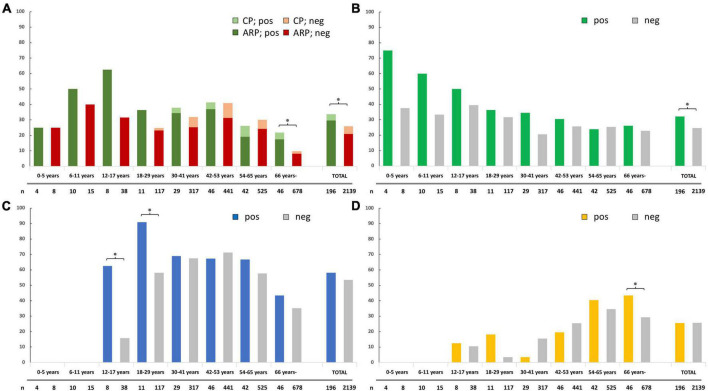
**(A)** Rate of acute recurrent pancreatitis (ARP) and chronic pancreatitis (CP) in different age groups of acute pancreatitis (AP) patients with positive and negative pancreatic family history; **(B)** rate of idiopathic etiology at time of the index AP registry enrolment; **(C)** rate of current alcohol consumption and/or smoking at the time of the index AP registry enrolment; and **(D)** rate of diabetes and/or hyperlipidemia at the time of the index AP registry enrolment. The * sign indicates a statistically significant difference between positive and negative pancreatic family history groups (<0.05). n, total number of participants with data on the examined variable; CP, chronic pancreatitis; ARP, acute recurrent pancreatitis; pos, positive pancreatic family history group; neg, negative pancreatic family history group.

Figure 1A shows the rate of ARP and CP (developed later or already diagnosed) with or without pancreatic family history categorized by the age of the index involvement in the AP registry. The higher rate of ARP was noted in childhood, even more so in the positive than the negative family history groups, but without statistical significance. Overall, a significantly higher rate of ARP and/or CP was found in the positive family history group (33.7 vs. 25.9%, *p* = 0.018). A figure not separating ARP and CP is available in our Supplementary material (Supplementary Figure 4).

### Association with idiopathic etiology, alcohol, smoking, and metabolic risk factors

Among patients with a negative pancreatic family history, the rate of idiopathic episodes was higher in children (30–40%) than in adults (20–30%). We found an excess of idiopathic etiology in children with a positive family history (75% 0–5 years, 60% 6–11 years), which decreased over time to meet the negative group. Statistically significant difference was found overall (32.1 vs. 24.6% in the positive vs. negative groups, respectively, *p* = 0.020; Figure 1B).

We found a significantly higher rate of current alcohol consumption and/or smoking at the index case in the positive family history group in ages 12–17 years (62.5 vs. 15.8%, *p* = 0.013) and 18–29 years (90.9 vs. 58.1%, *p* = 0.049) but not overall (58.2 vs. 53.4%, *p* = 0.204). In the remaining age groups, balanced distribution was found (Figure 1C).

A significant difference between positive and negative family history groups regarding the presence of diabetes mellitus (DM) and/or hyperlipidemia at the time of the index case was observed only in patients 66 years old or above (43.5 vs. 29.4%, respectively, *p* = 0.044) but not overall (25.5 vs. 25.7%, *p* = 0.950) or in any other age subgroup (Figure 1D).

**FIGURE 2:**
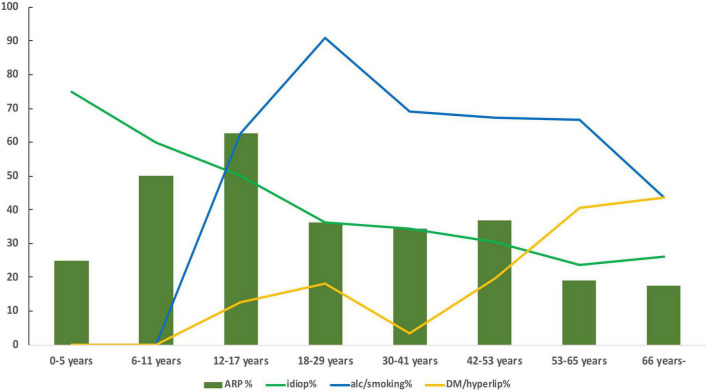
Pancreatitis recurrence rate (ARP%, dark green columns), idiopathic etiology rate (idiop%, light green line), alcohol and/or smoking prevalence (alc/smoking%, blue line), and diabetes and/or hyperlipidemia prevalence (DM/hyperlip%, yellow line) at the time of the index enrolment in the AP registry in the positive pancreatic family history group.

Figure 2 shows the recurrence rate and prevalence of discussed explanatory factors of familial aggregation in the positive pancreatic family history group to facilitate the interpretation of the above-presented results.

## Discussion

In our analysis, we evaluated ARP and CP rates and accompanying factors in different pediatric and adult age groups of AP, according to the presence of pancreatic family history.

Overall, we found a significantly higher rate of ARP or CP in the positive family history group. In the age-based subgroups, we observed a consistently higher rate of ARP or CP in the positive groups, but without statistical significance. The reason behind this was a relatively low number of participants in the pediatric subgroups and only subtle differences in the adult subgroups. It is likely that with higher patient numbers, the marked difference in the pediatric subgroups would be retained and statistical significance would be achieved, reflecting the available evidence. On the other hand, further increasing adult subgroups—while it could lead to significant results—would likely still be a clinically irrelevant difference. In our opinion, family history should not be used as a prognostic factor for recurrence and CP among adults.

The incidence rate of ARP peaked in those who had their index episode between 6 and 17 years, the highest percentage difference between positive and negative pancreatic family history groups was noted between 12 and 17 years.

While the negative family history group had a rate of idiopathic etiology consistently in the 20–40% range, the positive group had an excess of idiopathic AP in the pediatric age groups: peaking at 75% at 0–5 years then steadily decreasing to meet the negative group in adulthood. This is likely due to genetic risk factors being responsible for familial aggregation among pediatric patients, especially in early childhood. No differences in adults are in line with the findings of Jalaly et al. who performed genetic testing in 134 adults with idiopathic AP and found that family history does not predict pathogenic variants (16).

However, next to the decline of differences in idiopathic etiology, another factor emerged at 12–17 years; we found a significantly higher rate of alcohol consumption and/or smoking in patients with a positive pancreatic family history, who had their index episode in this, or the following age group (18–29 years). The most likely explanation is the well-documented association between parental and offspring alcohol consumption: a systematic review found that in 12 out of 12 included studies, parents’ drinking was predictive of adolescents’ alcohol use (17), and a cross-sectional study of 982 adolescents found hazardous paternal drinking to be strongly associated (OR = 2.90) with use (18). Contrary to the seemingly similar rationale, empirical evidence does not support the association between parental and adolescent smoking (19, 20).

Regarding DM and hyperlipidemia, metabolic risk factors for AP (21, 22), we found low prevalence in pediatric patients, in conformity with low childhood prevalence reported in the literature, 1.93/1,000 for type 1 DM, 0.46/1,000 for type 2 (23), and 2–4/1,000 for familial hypercholesterolemia (24–26). With the onset of childhood obesity, most prominently from early adolescence, the prevalence of type 2 DM and hyperlipidemic states start to rise, transitioning to the higher rate seen among adults: for DM, around 40–130/1,000 in the general adult population and 170–250/1,000 above 65 years (27–30). We expected to see significant differences or at least a tendency favoring the positive pancreatic family history group since metabolic syndrome and DM both have genetic and learned behavioral components that could lead to their accumulation in the family (31). We only noted such difference above 66 years, with a tendency starting to show in the 54–65 years’ subgroup.

The prevalence rates of alcohol consumption, smoking, DM, and hyperlipidemia are over-represented in our cohort as compared to the general population. Quite understandably, these are all likely to accumulate in a cohort of patients with AP, as risk factors for the disorder.

### Strengths and limitations

To our knowledge, this was the first cohort study to examine the ARP and CP prognostic role of family history in adults and the first cohort representing both pediatric and adult patients seeking associations between pancreatic family history and clinical factors that could be in the background of this familial aggregation. One of the main strengths of this study is that the participants come from multiple centers, countries, and continents, signifying wide representativeness. We applied a uniform data collection, following the same structure in all ages, thus enhancing the comparability of adult and pediatric populations. Our patient enrolment encompassed a period of 8 years and the index case in the registry is not necessarily the first AP of the participant—thus, we believe that our conclusions regarding the ARP rate are valid.

Conclusions regarding CP rate, however, should be handled with caution since they are probably under-represented, especially in the pediatric age groups. Another limitation of this study is that, even though in proportion to the enrolled adults, the number of pediatric patients is appropriate, it is still relatively low, while we observed the tendencies in ARP, idiopathic etiology, and exogenous risk factors that we expected, these associations were not backed up by statistical significance due to low event numbers. It should also be stated that the first AP episode enrolled in our registry is not necessarily the first episode of the individual—although it was in most cases. We performed our analyses this way since our data of interest could not be gathered for non-enrolled episodes without a high possibility of bias. In addition, though our intent was to examine family history in a purely clinical context, and idiopathic etiology tendency matched our expectations, it is only a surrogate marker—genetic analysis of all patients would have clarified genetic background; this was currently beyond our scope.

### Implications

Positive family history most likely signifies genetic background in early childhood. During adolescence and early adulthood, alcohol consumption and smoking emerge—clinicians should be aware of the significant association with pancreatic family history (probably due to harmful consumption in the family) and consider targeted intervention in such cases. Our analysis revealed that contrary to current viewpoints, positive pancreatic family history is not a prognostic factor for ARP and CP in adults, so it should not be used as such.

## Data availability statement

The raw data supporting the conclusions of this article will be made available by the authors, without undue reservation.

## Ethics statement

The studies involving human participants were reviewed and approved by Scientific and Research Ethics Committee of the Medical Research Council (Hungary). Written informed consent to participate in this study was provided by the participants’ legal guardian/next of kin.

## Author contributions

MJ, AP, and PH formulated the original concept. AS, AW, AN, NL, AT, IT, ÁV, BE, FI, LC, MP, and NF contributed to this concept. MJ, AP, PH, AS, AW, AN, NL, AT, IT, ÁV, BE, FI, LC, and MP took part in data acquisition. NF and MJ conducted the analyses. MJ and AP drafted the manuscript. PH, AS, AW, AN, NL, AT, IT, ÁV, BE, FI, LC, MP, and NF revised it critically for intellectual content. All authors have read and approved the final version of the manuscript.
