# Supplementary material for: Pancreatic family history does not predict disease progression but connotes alcohol consumption in adolescents and young adults with acute pancreatitis: Analysis of an international cohort of 2,335 patients
Source: Front Med (Lausanne). 2022 Sep 12;9:801592. doi: 10.3389/fmed.2022.801592 (PMC9511134; doi:10.3389/fmed.2022.801592)
Supplement: Supplementary file 1 [file Data_Sheet_1.pdf]

Supplementary material

Manuscript title: Pancreatic family history doesn’t predict disease progression, but connotes alcohol consumption in adolescents and young adults with acute pancreatitis: Analysis of an international cohort of 2,335 patients.

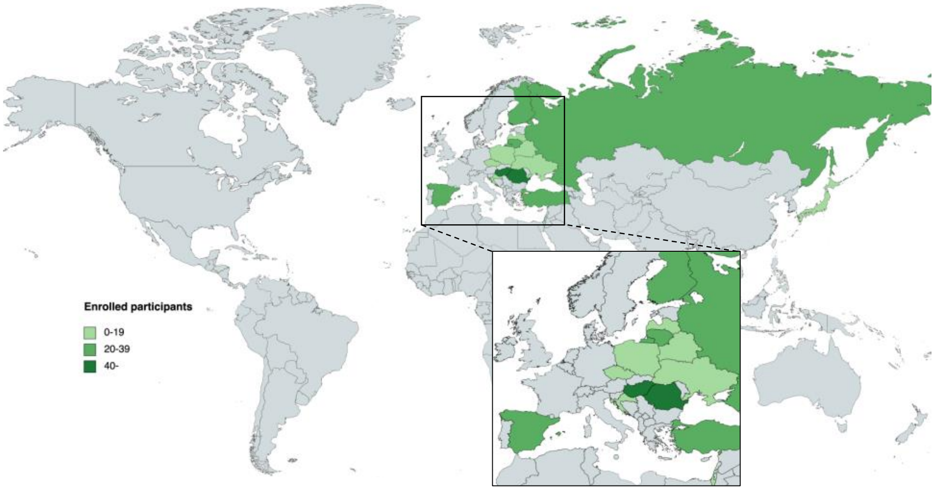

Country	n
Hungary	2079
Romania	63
Lithuania	31
Russia	28
Spain	28
Finland	25
Turkey	20
Poland	12
Croatia	11
Czech Republic	11
Belarus	8
Ukraine	8
Latvia	6
Israel	3
Japan	2
	2335

Supplementary Figure 1: Distribution of participant enrolment in countries involved. n: number of enrolled participants.

Country	City	Institution	n
Belarus	Gomel	Gomel Regional Clinical Hospital	8
Croatia	Rijeka	Clinical Hospital Center Rijeka	11
Czech Republic	Ostrava	Vítkovice Hospital	11
Finland	Helsinki	Helsinki University Central Hospital	25
Hungary	Békéscsaba	Dr. Réthy Pál Hospital	67
	Budapest	Bethesda Children’s Hospital	3
		Bajcsy-Zsilinszky Hospital	113
		Buda Hospital of the Hospitaller Order of Saint John of God	6
		First Department of Pediatrics, Semmelweis University	9
		Pál Heim National Pediatric Institute	10
		Second Department of Internal Medicine, Semmelweis University	2
		Second Department of Pediatrics, Semmelweis University	3
	Debrecen	Department of Internal Medicine, University of Debrecen	165
		Department of Surgery, University of Debrecen	5
	Gyula	Pándy Kálmán Hospital of County Békés	31
	Kecskemét	Bács-Kiskun County University Teaching Hospital	10
	Kiskunhalas	Kiskunhalas Semmelweis Hospital	1
	Makó	Healthcare Center of County Csongrád	10

	Miskolc	Borsod-Abaúj-Zemplén County Hospital and University Teaching Hospital	19
	Pécs	Department of Pediatrics, University of Pécs	9
		First Department of Medicine, University of Pécs	794
	Szeged	Albert Szent-Györgyi Clinical Center of Pediatrics and Child Health Centre	8
		First Department of Medicine, University of Szeged	299
		Second Department of Medicine, University of Szeged	81
		Department of Anaesthesiology and Intensive Therapy, University of Szeged	12
	Székesfehérvár	Szent György University Teaching Hospital of County Fejér	380
	Szentes	Dr. Bugyi István Hospital	21
	Szombathely	Markusovszky University Teaching Hospital	19
	Zalaegerszeg	St. Rafael Hospital of Zala County	2
Israel	Jerusalem	Hadassah Hospital	3
Japan	Tokyo	Keio University	2
Latvia	Riga	Pauls Stradiņš Clinical University Hospital	6
Lithuania	Vilnius	Vilnius University Hospital Santariskiu Klinikos	31
Poland	Kraków	Jagiellonian University Medical College	12
Romania	Bucharest	Central Military Emergency Hospital "Dr Carol Davila"	1
		Grigore Alexandrescu Children`s Hospital, Carol Davila University	8
	Targu Mures	Mures County Emergency Hospital	54
Russia	St. Petersburg	Saint Luke Clinical Hospital	28
Spain	Sant Pere de Ribes	General Surgery, Consorci Sanitari del Garrof	28
Turkey	Istanbul	Hospital of Bezmialem Vakif University, School of Medicine, Istanbul	20
Ukraine	Kiev	Bogomolets National Medical University	8
		**Total number of participants**	**2335**

Supplementary Table 1: Distribution of centers and enrolled participants. n: number of participants

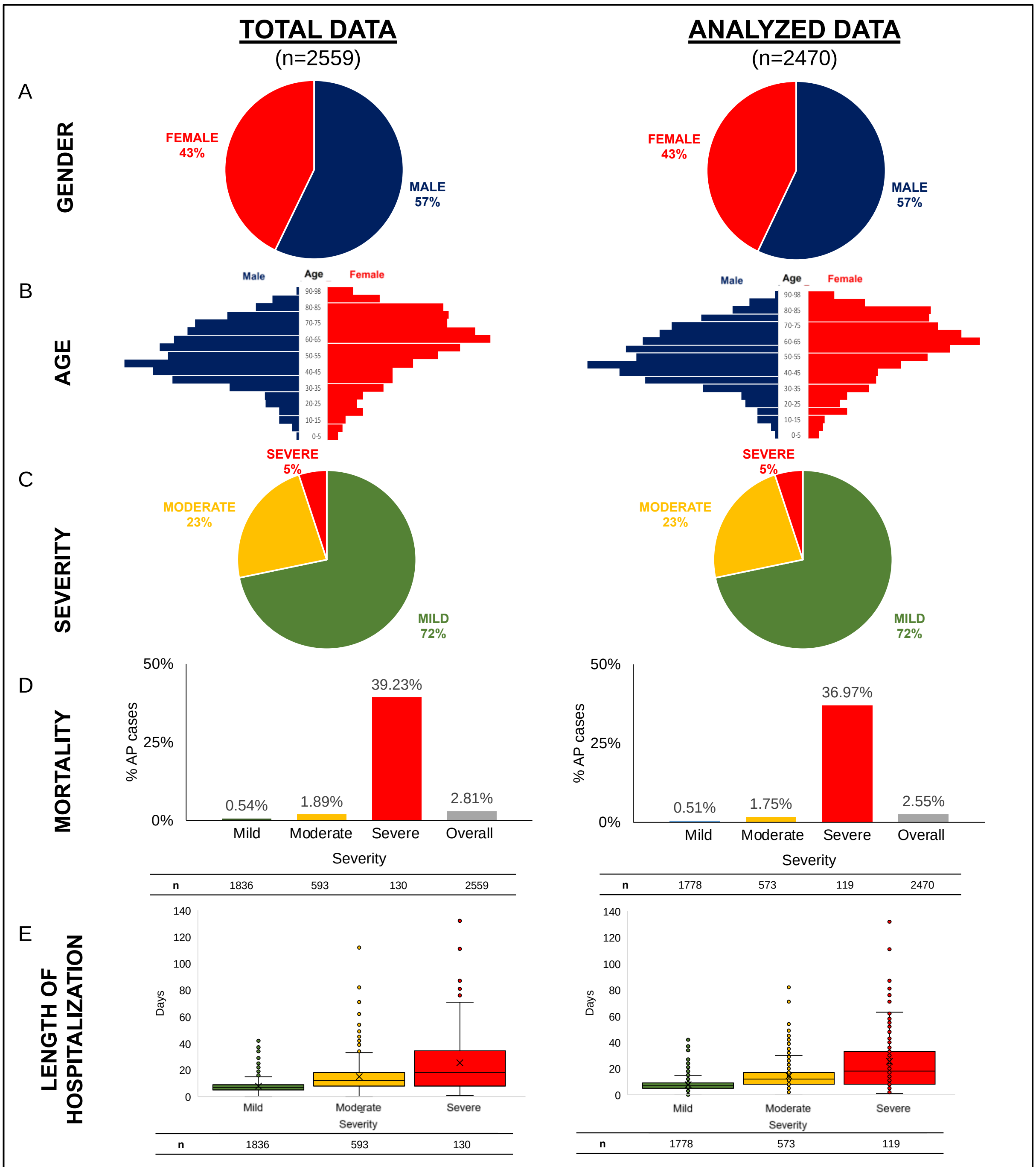

F

ETIOLOGY

Etiology	n	%
Biliary	988	39
Alcohol	502	20
Hypertriglyceridemia	85	3
Biliary + alcohol	40	2
Biliary + hypertriglyceridemia	12	0
Alcohol + hypertriglyceridemia	60	2
Post-ERCP	72	3
Drug induced	19	1
Idiopathic	602	24
Other	179	7
	2559	100

Etiology	n	%
Biliary	957	39
Alcohol	485	20
Hypertriglyceridemia	82	3
Biliary + alcohol	39	2
Biliary + hypertriglyceridemia	12	0
Alcohol + hypertriglyceridemia	60	2
Post-ERCP	72	3
Drug induced	19	1
Idiopathic	576	23
Other	168	7
	2470	100

Supplementary Figure 2: Representativeness. We compared the enrolled AP cases in our examined cohort (those with data on the presence of pancreatic family history) to an average AP population (all the patients in the AP registry). Our cohort was representative in terms of gender, age, AP severity, mortality, length of hospitalization and AP etiology – quite understandably, since almost all patients had data on the presence of pancreatic diseases in their respective families. n: total number of participants with data on the examined variable

**EPIDEMIOLOGY, ETIOLOGY**	**OVERALL**	**UPLOADED DATA**	**%**
Age	2470	2470	100
Gender	2470	2470	100
Etiology	2470	2470	100
Average uploaded data	*7410*	*7410*	*100*

**PERSONAL AND FAMILY MEDICAL HISTORY**	**OVERALL**	**UPLOADED DATA**	**%**
Acute pancreatitis in the personal history	2470	2470	100
Number of previous episodes among recurrent cases	517	483	93
Chronic pancreatitis in the personal history	2470	2470	100
History of diabetes mellitus	2470	2466	100
History of non-transient dyslipidemia	2470	2118	86
Family history of pancreatic diseases	2470	2470	100
Average uploaded data	*12867*	*12477*	*97*

**RISK FACTORS**	**OVERALL**	**UPLOADED DATA**	**%**
Alcohol consumption	2470	2464	100
Smoking	2470	2464	100
Average uploaded data	4940	4928	*100*

**OUTCOMES**	**OVERALL**	**UPLOADED DATA**	**%**
Severity (mild/moderately severe/severe)	2470	2470	100
Mortality	2470	2470	100
Average uploaded data	4940	*4940*	*100*
TOTAL	***30157***	**29755**	***99***

Supplementary Table 2: Data quality

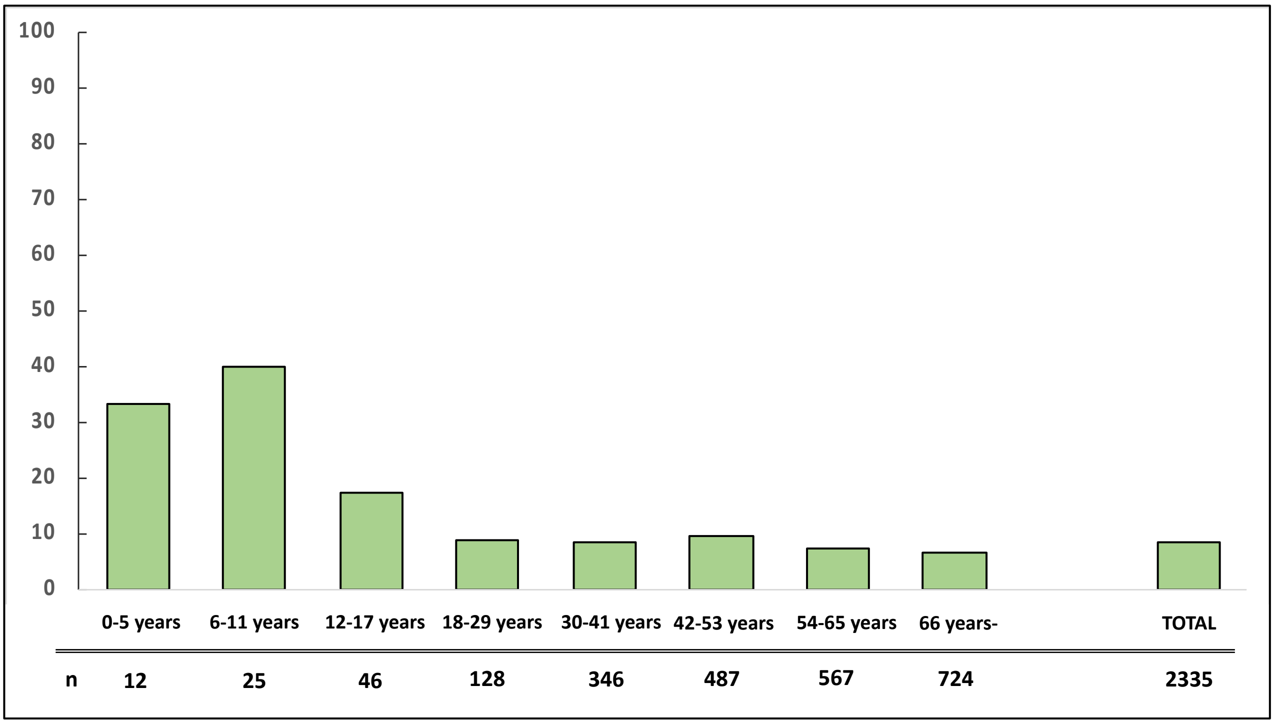

Supplementary Figure 3: Rate of participants with positive pancreatic family history in each age subgroup. n: total number of participants with data on the examined variable.

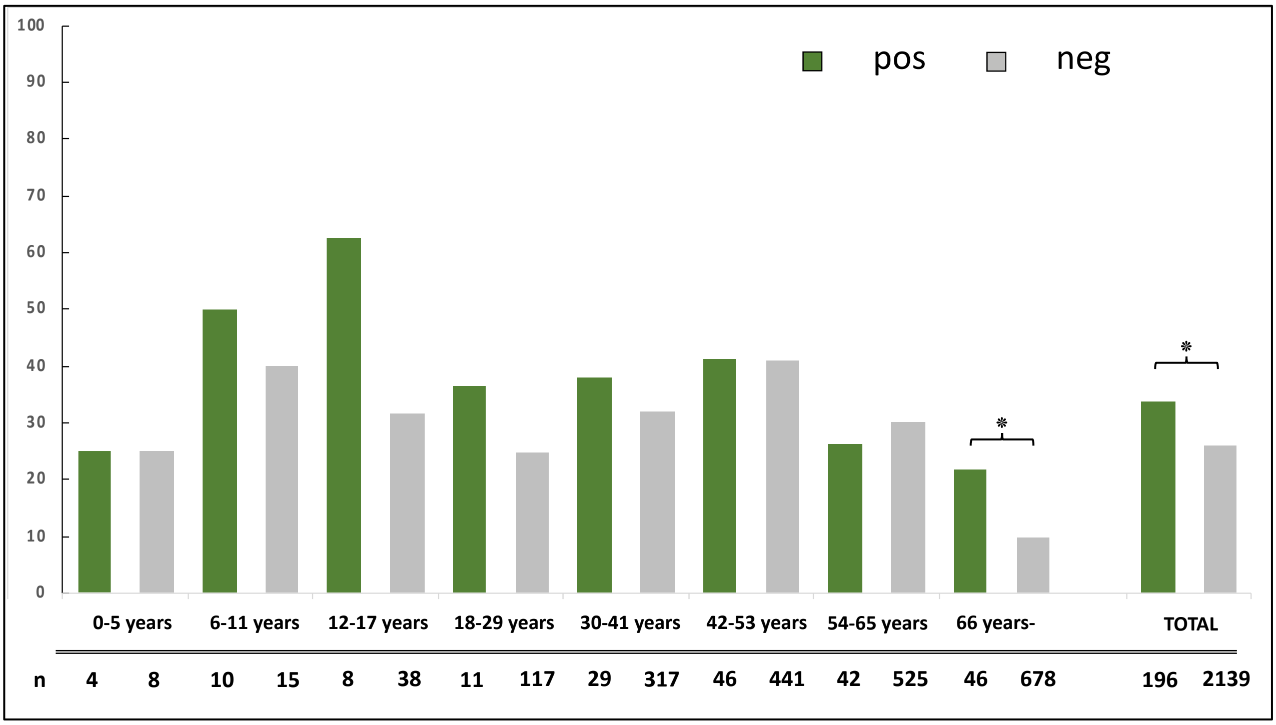

Supplementary Figure 4: Height of bars indicate the rate of acute recurrent pancreatitis and/or chronic pancreatitis in acute pancreatitis patients with positive and negative pancreatic family history, according to their age at the time of the index enrolment in our registry. Star sign indicates statistically significant difference between positive and negative pancreatic family history groups (<0.05). n: total number of participants with data on the examined variable; pos: positive pancreatic family history group; neg: negative pancreatic family history group.
